# Impact of surgical resection of butterfly glioblastoma on survival: a meta-analysis based on comparative studies

**DOI:** 10.1038/s41598-021-93441-z

**Published:** 2021-07-06

**Authors:** Rafał Chojak, Marta Koźba-Gosztyła, Katarzyna Słychan, Daniel Gajos, Marek Kotas, Michał Tyliszczak, Bogdan Czapiga

**Affiliations:** 1 Faculty of Medicine, Wrocław Medical University, Ludwika Pasteura 1, 50-367 Wrocław, Poland; 2 Department of Neurosurgery, 4th Military Hospital in Wrocław, Wrocław, Poland; 3 Department of Nervous System Diseases, Faculty of Health Sciences, Wrocław Medical University, Wrocław, Poland

**Keywords:** CNS cancer, Outcomes research

## Abstract

Butterfly glioblastoma (bGBM) is a rare brain tumor that invades both hemispheres by crossing the corpus callosum. bGBM is associated with a dismal prognosis with a median survival time of a few months. Surgical resection is a rare treatment option due to the unfavorable location and assumed poor risk-to-benefit ratio. Therefore, a biopsy-alone approach is considered the main treatment option. This meta-analysis aimed to systematically evaluate whether resection of bGBM is associated with improved overall survival compared with biopsy alone. We searched three databases to find studies that compare resection with biopsy in 6-, 12- and 18-months overall survival in patients with bGBM. We calculated the pooled relative risk (RR) of mortality using a random-effects model. Five studies with 194 patients were included in the meta-analysis. Mortality was decreased for resection compared with biopsy at 6-months (RR 0.63 [95% CI 0.44–0.91]). No significant differences in overall survival were found at 12 (RR 0.76 [95% CI 0.50–1.14]) and 18-months (RR 0.84 [95% CI 0.56–1.26]). Surgical resection of bGBM is associated with an improved 6-months overall survival compared with biopsy alone. We have not found strong evidence supporting the superiority of resection over biopsy alone in overall survival at 12 and 18-months.

## Introduction

Butterfly glioblastoma (bGBM) is a rare type of glioblastoma, a brain tumor that invades both hemispheres by crossing the corpus callosum, deriving its name from the shape of patterns it forms in MRI images. bGBM is associated with a poor prognosis with a median survival of 3.3–6 months^1–3^, and only 9% of patients with bGBM survive 2-years^4^.

The current management options of newly diagnosed glioblastoma are surgical resection or biopsy, followed by radio- and/or chemotherapy^5^. Due to unfavorable location and assumed poor risk-to-benefit ratio of resection, a biopsy is the preferred treatment option. However, there is growing evidence for the benefits of resection of these tumors^6^. Studies comparing survival time after resection versus biopsy alone in the management of bGBM are limited, often with small sample sizes. Therefore, the choice between resection or biopsy is mostly based on individual experience.

This meta-analysis aimed to systematically evaluate whether resection of bGBM is associated with improved 6-, 12- and 18-months overall survival compared with biopsy alone.

## Methods

### Overview

The meta-analysis was conducted according to the preferred reporting items for systematic reviews and meta-analyses (PRISMA) guidelines and recommendations^7^.

### Search strategy

We searched the Web of Science, PubMed, and Scopus for English-language articles reporting data relevant to the meta-analysis. Databases were searched without date restrictions; we finished searching in March 2021. The key terms were related to glioblastoma, butterfly, and corpus callosum.

Three researchers (KS, MT, and DG) independently screened all titles and abstracts for their suitability. The full texts of potentially relevant articles were retrieved for detailed eligibility assessment according to selection criteria. Any discrepancies during the selection and extraction process were resolved by discussion and consensus. Furthermore, we hand-searched bibliographies of included articles and related reviews to identify additional articles relevant to the meta-analysis.

### Selection criteria

Our inclusion criteria were studies that: (1) compared biopsy with resection in patients with bGBM; (2) reported data on 6- and/or 12- and/or 18-months overall survival. We also applied the following exclusion criteria: (1) review articles, conference abstracts, letters to editors, case studies; (2) studies with irrelevant data; (3) duplicate publication; (4) cadaveric studies; (5) animal studies; (6) sample size less than 5 patients per study arm.

### Data extraction

Two reviewers (DG and RC) independently extracted data from included articles into a spreadsheet using Microsoft Excel (2010; Microsoft Corporation, Redmond, WA, USA). We recorded: (a) the number of patients who were alive at 6-, 12-, and 18-months follow-up; (b) the first author’s last name; (c) year of publication; (d) country of a study performed; (e) enrollment dates; (f) numbers of patients with bGBM; (g) sex; (h) age; (i) preoperative tumor volume; (j) use of adjuvant therapy; (k) IDH1/2 mutation and MGMT methylation status.

### Quality assessment

We used the Newcastle Ottawa Scale to assess the quality of studies included in the meta-analysis^8^. We carried out quality assessments individually, then one of us (RC) compared it. The NOS ranges between zero (highest risk for bias) up to nine points (lowest risk for bias). Studies with scores of ≥ 6 were considered as high-quality.

### Outcomes

The primary study outcomes were differences in 6-, 12- and 18-months mortality between patients who had resection compared with biopsy alone. The secondary outcomes were differences in preoperative tumor volume, administered adjuvant therapy, IDH1/2 mutation, and MGMT methylation status in these groups.

### Statistical analysis

Selected characteristics of the included studies were summarized by treatment group, as means or medians, and standard deviation (SD), ranges or interquartile ranges for continuous variables, and as percentages and numbers for categorical variables. Median values were converted to mean with SD to facilitate computations^9^, missed SD was calculated from the *P*-value^10^. We used *t* test and Pearson’s χ^2^ test to compare continuous and binary data, respectively. Values of *P* < 0.05 were considered statistically significant.

The meta-analysis was performed to calculate pooled relative risks (RRs) with 95% confidence intervals (CIs) of mortality of the resection versus a biopsy alone. A Mantel–Haenszel method for RR with DerSimonian and Laird random-effects model was applied^11^. We used a conservative Knapp–Hartung modification, as is recommended in meta-analyses with few included trials^12^. We used Cochrane’s Q test and the I^2^ statistic to evaluate heterogeneity. A Cochrane's Q *P*-value of < 0.10 indicated significant heterogeneity. I^2^ represents the percentage of total variation across studies, the I^2^ statistic value greater than 50% suggests substantial heterogeneity^13^. To probe the source of heterogeneity, we did post-hoc sensitivity analyses. Results are presented in a forest plot with 95% CI. All analyses were done with the use of RStudio (version 1.3.1093).

## Results

### Search results

**Figure 1:**
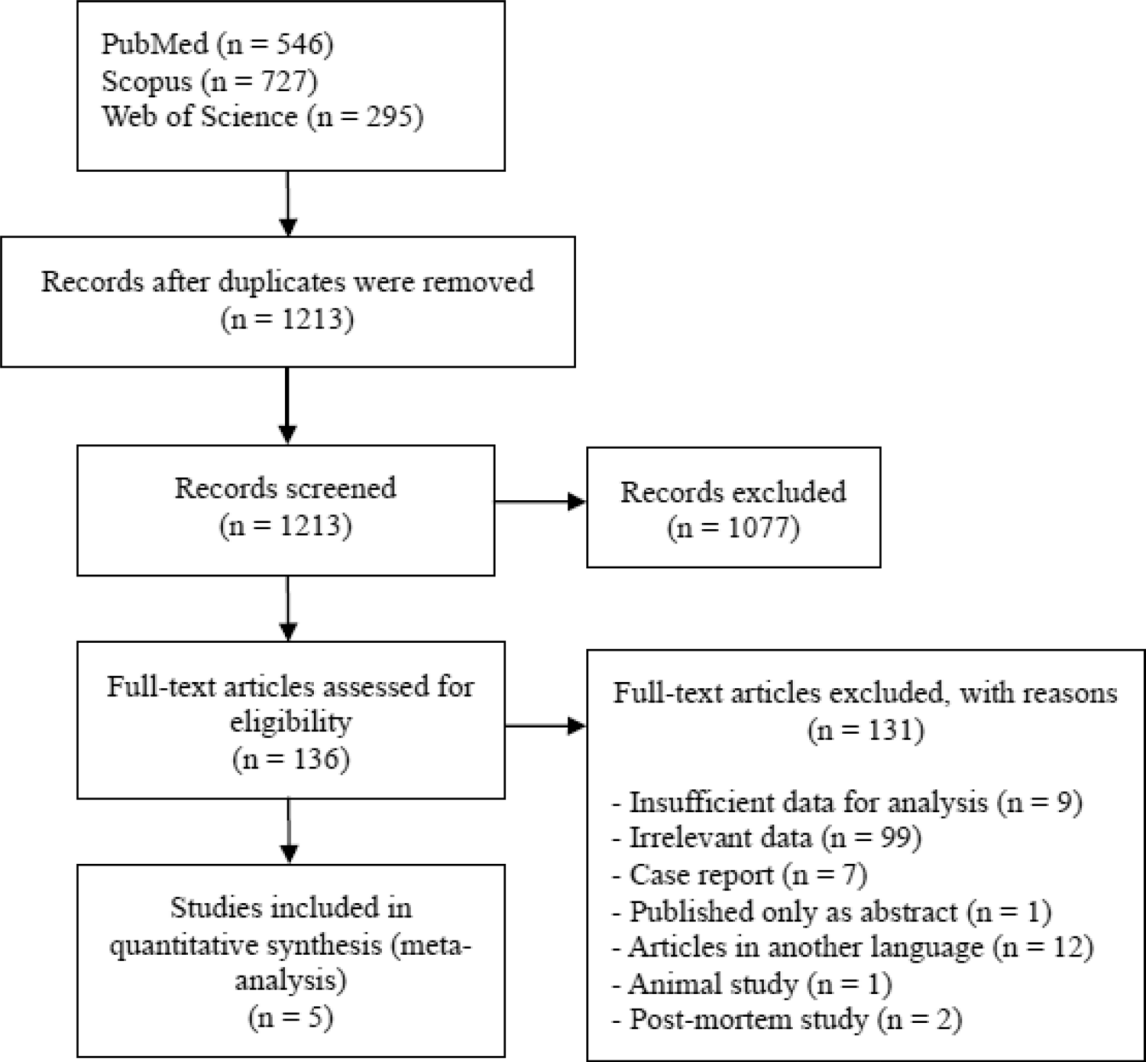
Flow chart showing search strategy.

We identified 1628 records by database searching, 415 duplicates were removed. All titles and abstracts were screened for potentially relevant data. Of these, 136 full-text articles were assessed for their suitability. Finally, 5 articles^2–4,14,15^ that included 194 patients treated for bGBM were included. Eighty-eight patients had a resection of the tumor, and 106 had a biopsy alone. All included studies were retrospective cohort studies. The process of study identification is shown in Fig. 1. Studies and population characteristics are shown in Table 1.

**Table 1:** Characteristics of included studies. *N* number of patients, *NOS* Newcastle–Ottawa Scale score, *IQR* interquartile range, *SD* standard deviation, *Median.

Study	Country	Recruitment period	N	NOS	Resection			Biopsy		
					N	Mean age	Sex distribution (male; female)	N	Mean age	Sex distribution (male; female)
Franco et al. 2020	Germany	2005–2017	55	7	25	62.3* (IQR 50.5–66.9)	60%, 40%	30	68.5* (IQR 62.1–76)	63%, 37%
Chaichana et al. 2014	US	2007–2012	48	8	29	61.7 (SD 12.8)	48%, 52%	19	54.2 (SD 17.9)	58%, 42%
Dayani et al. 2018	US	2004–2014	39	8	14	51.2 (range 20–83)	71%, 29%	25	63.9 (24–80)	52%, 48%
Dziurzynski et al. 2012	US	2000–2010	23	7	11	NA	NA	12	NA	NA
Opoku-Darko et al. 2017	Canada	2004–2016	29	8	9	56.9 (SD 3.7)	67%, 33%	20	61.1 (SD 2.8)	60%, 40%

### Patients’ characteristic

All of the included studies, except one, reported the age of patients. The mean age ranged from 51.2 to 62.3 years in the resection group, and from 54.2 to 68.5 years in the biopsy group. Adjuvant therapy was received by 67.8% and 51.7% of patients in the resection and the biopsy group, respectively (*P* = 0.054). Preoperative tumor volume was reported in all studies. The mean preoperative tumor volume was 55.2 cm^3^ in the resection group and 44.8 cm^3^ in the biopsy group (*P* = 0.023). The IDH1/2 mutation and MGMT methylation status were available in three studies^3,4,14^ that reported it in 77 and 43 cases, respectively. There were no significant differences in the genetic profiles between the resection and biopsy groups. Comparisons of preoperative tumor volume, frequency of adjuvant treatment, and genetic profile between groups are presented in Table 2.

**Table 2:** Preoperative tumor volume, adjuvant therapy, and genetic profile comparison between groups. *N* number of patients, *SD* standard deviation.

	Resection	Biopsy	*P*-value
Mean preoperative tumor volume in cm^3^ (SD)	55.2 (35.5)	44.8 (27.7)	0.0229
Adjuvant therapy (N)	67.8% (40/59)	51.7% (45/87)	0.0541
IDH1/2 Mutation (N)	0.0% (0/37)	2.5% (1/40)	0.3362
MGMT methylation (N)	34.8% (8/23)	60.0% (12/20)	0.1022

### 6-Months survival

**Figure 2:**
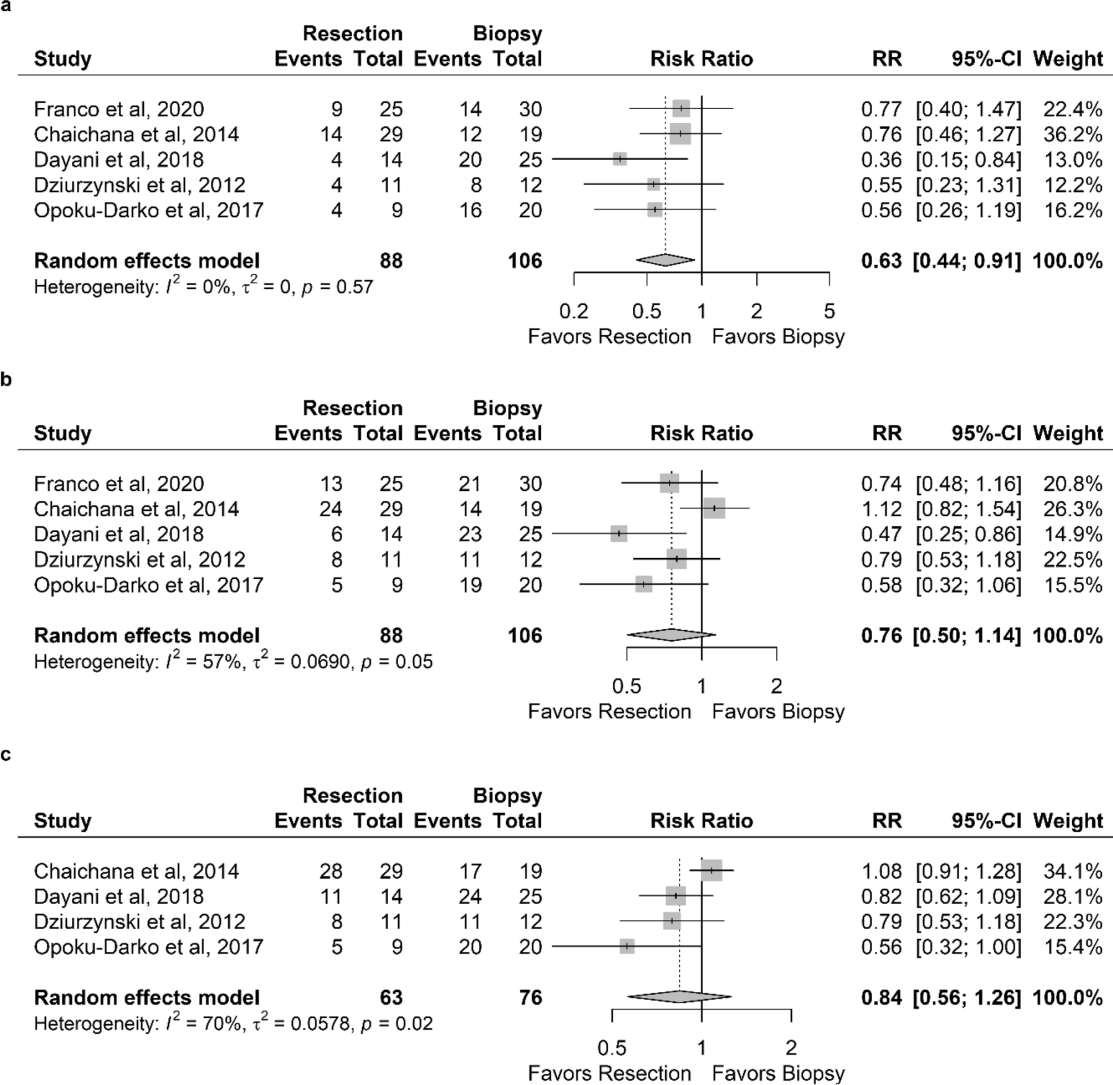
Forest plots showing the relative risk (RR) for (**a**) 6, (**b**) 12, and (**c**) 18-months mortality for resection versus biopsy alone.

All studies reported overall survival at 6-months (see Fig. 2a). The pooled estimates showed a substantially improved overall survival after resection compared with a biopsy alone at 6-months (RR 0.63 [95% CI 0.44–0.91]).

### 12-Months survival

All studies reported overall survival at 12-months (see Fig. 2b). No significant differences were found for the 12-months survival (RR 0.76 [95% CI 0.50–1.14]).

### 18-Months survival

All but one study reported overall survival at 18-months (see Fig. 2c). No significant improvement in the overall survival was found for resection compared with a biopsy alone (RR 0.84 [95% CI 0.56–1.26]).

### Heterogeneity and sensitivity analysis

We observed a substantial heterogeneity between the datasets for the 12- and 18-months outcomes (I^2^ = 57% and I^2^ = 70%, respectively). Therefore, we performed sensitivity analyses excluding each study individually and recalculating the RRs and 95% CI. The heterogeneity for the RR outcome at 12- and 18-months was reduced to I^2^ = 0%, and the RR outcome at 12-months became significant in favor of resection (RR 0.68 [95% CI 0.48–0.97]) after excluding the study of Chaichana et al.^15^. No evidence of heterogeneity between studies at the 6-months outcome was observed.

### Quality assessment

The mean NOS score was 7.6 (standard deviation: 0.55) indicating that the methodological quality was generally good (Table 1). No study was excluded due to a low-quality score.

## Discussion

According to our knowledge, this is the first meta-analysis that examines the association between the treatment strategy (resection vs biopsy) and survival of patients with diagnosed bGBM. It demonstrated that resection could significantly reduce the 6-months mortality rate compared with biopsy alone. We did not find any significant differences in the survival rate at 12- and 18-months comparing a resection of the tumor with a biopsy alone. These results should be interpreted with caution due to observed moderate heterogeneity.

The study of Dayani et al.^14^ showed the highest risk reduction in favor of resection This might be due to significant differences in patients characteristics between biopsy and resection groups. Biopsy patients were significantly older, and their median Karnofsky Performance Score (KPS) was 20 points lower (*P* = 0.003) in comparison with the resection cases. There was no such difference between other included studies^2–4,15^. These factors might have favored the resection group since both age and KPS are associated with survival in GBM patients^16,17^. Therefore, in a sensitivity analysis, we excluded this study, but this did not significantly change the main findings. A multivariate regression analysis performed by Dayani et al.^14^ showed that resection impacted survival independently of both age and KPS.

The only study that favored a biopsy at 12 and 18-months was Chaichana et al.^15^. It was also the only study where the biopsy cohort was younger than the resection one. Matching groups for a number of factors—including age, KPS, tumor size, and adjuvant therapy—increased the difference in survival between groups (7.0 vs. 3.5 months, *P* = 0.03 in matched-pair analysis; 6.4 vs. 4.2 months in the whole cohort). In the matched groups, the 6- and 12-months survival rates were 60% and 12% for resection, and 12% and 0% for biopsy alone, respectively. By the exclusion of this study, the main findings changed significantly in favor of resection at the 12-months outcome. Moreover, between-study heterogeneity was significantly reduced when this study was excluded. Therefore, the results of this study should be interpreted with caution.

Only 4% of patients with GBM survive 5 years^18^, and there is a lack of studies reporting 5-year survival of patients with bGBM. In the study of Dziurzynski et al.^2^, the longest survival length was 1018 days (2.8 years). Finneran et al.^19^ described a patient with bGBM who survived more than 5 years from the initial diagnosis.

Resection of bGBM is challenging, and special attention needs to be paid to avoid injury of the main vascular and subcortical structures during tumor debulking. However, resection with low persistent neurological deficits and without long-term decline is possible. The use of neuronavigation and subcortical mapping might improve safety and outcomes^6,14^. Sughrue^20^ described the key points in resecting of the anterior, the middle callosum, and the splenial bGBM. Burks et al.^21^ presented evidence for the safe removal of anterior bGBM during awake brain surgery. Forster et al.^6^ concluded that the benefit of tumor resection might outweigh morbidity in patients with a preoperatively good neurological state. Moreover, the quality of life in patients who had a resection was improved compared with biopsy alone. The higher extent of resection is also associated with improved QOL^22,23^.

This meta-analysis has some limitations. First, we searched only for articles in English, which carries a risk of omitting eligible studies published in other languages. Second, it consists of only a few studies that might cause overly narrow confidence intervals; therefore, we applied the Hartung–Knapp conservative modification. Third, the resection and the biopsy cohorts were not adjusted for preoperative tumor volume and adjuvant therapy use. The number of patients who received adjuvant therapy was higher in the resection cohort compared with the biopsy one. This might have favored the resection group in terms of survival and potentially confounded the results. Many other prognostic factors such as tumor-related gene expression patterns, immune-related tumor micro-environment,the extent of tumor resection, performance status, nutritional status, and mutations (e.g. IDH1/2 mutation or MGMT methylation) could not have been reliable investigated and controlled for, mainly due to lack of data^24–29^. Stratifying outcomes based upon these factors should be addressed in further studies. Ideally, subgroup analysis by age, sex, ethnicity, and other factors should also be performed^30–32^. Because of these potential confounders and the fact that all included studies were single-center retrospective cohort studies, with all their potential limitations, we can only report an association (of surgical resection on survival rate) rather than causation. The potential causal effects should be controlled for in further studies with, for example, the use of the Mendelian Randomization analysis^33–35^. Lastly, there might be a general publication bias due to unpublished studies with negative findings, which might have led to biased results. We have not assessed publication bias due to a low number of included studies since it may lead to inappropriate and misleading findings when there are less than 10 studies included in the meta-analysis^36,37^.

## Conclusions

Our findings suggest that surgical resection is associated with an improved 6-months survival rate in patients diagnosed with bGBM, as compared with biopsy alone. We have not found significant differences in survival rate at 12 and 18-months between resection and biopsy. In every case, an individual approach assessing the risk-to-benefit ratio of resection is necessary. There is also a need to develop new treatment strategies that will prolong survival in patients diagnosed with bGBM. Results of this meta-analysis should be interpreted with caution due to the retrospective nature of included studies. In the future, a more precise machine-learning model can be constructed to predict the impact of surgical resection of butterfly glioblastoma on survival when evaluating additional genomic and clinical information, including immune environment, genomic mutations, RNA, and protein modifications, and family history^38,39^.

## Data Availability

The datasets generated and/or analyzed during the current study are available from the corresponding author on reasonable request.
